# Embryonic Stem Cell-Derived Endothelial Cells for Treatment of Hindlimb Ischemia

**DOI:** 10.3791/1034

**Published:** 2009-01-23

**Authors:** Ngan F. Huang, Hiroshi Niiyama, Abhijit De, Sanjiv S. Gambhir, John P. Cooke

**Affiliations:** Division of Cardiovascular Medicine, Stanford University School of Medicine; Department of Radiology, Stanford University School of Medicine

## Abstract

Peripheral arterial disease (PAD) results from narrowing of the peripheral arteries that supply oxygenated blood and nutrients to the legs and feet, This pathology causes symptoms such as intermittent claudication (pain with walking), painful ischemic ulcerations, or even limb-threatening gangrene. It is generally believed that the vascular endothelium, a monolayer of endothelial cells that invests the luminal surface of all blood and lymphatic vessels, plays a dominant role in vascular homeostasis and vascular regeneration. As a result, stem cell-based regeneration of the endothelium may be a promising approach for treating PAD.

In this video, we demonstrate the transplantation of embryonic stem cell (ESC)-derived endothelial cells for treatment of unilateral hindimb ischemia as a model of PAD, followed by non-invasive tracking of cell homing and survival by bioluminescence imaging. The specific materials and procedures for cell delivery and imaging will be described. This protocol follows another publication in describing the induction of hindlimb ischemia by Niiyama et al.^1^

**Figure:**
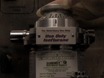


## Protocol

### 1. Differentiation of murine ESCs into endothelial cells

The protocol for differentiating ESCs into endothelial cells is described elsewhere and is not the focus of this protocol^2,3^. Briefly, the cells are allowed to differentiate, and the cells that are positive for the endothelial markers such as CD31 or vascular endothelial cadherin (VE-cadherin) are then purified by fluorescence activated cell sorting (FACS).

### 2. Construction of the Double Fusion Reporter Gene and Lentiviral Transduction

Bioluminescence can be used to track cells that are modified to express reporter genes such as firefly luciferase (fluc). For this application, our cells contain both fluc and enhanced green fluorescence protein (eGFP) fusion gene under the control of an internal ubiquitin promoter. In order to modify the cells, the transgene lentiviral vector containing key optimized genetic elements to fulfill bio-safety criteria as well as increased transduction efficiency, pFU-FG vector developed in our lab carrying the fluc-eGFP fusion reporter gene can be used to stably transduce the cells after differentiation. This fusion construct enables both bioluminescence and fluorescence tracking of the transplanted cells. The procedure for generating virus particles, transduction and production of labeled cells that express reporter genes is described elsewhere^4^.

### 3. Transplantation of ESC-derived endothelial cells to the ischemic hindlimb

Begin the procedure by preparing a mouse that has undergone hindlimb ischemia for cell transplantation. To do this, place the mouse into the anesthesia induction chamber containing 1–3% isoflurane in 100% oxygen at a flow rate of 1L/min. 
Leave the mouse in the induction chamber until it is unresponsive to external stimuli. Then remove the animal from the induction chamber.
Then, place the animal in the supine position onto the operating table and connect it to a continuous flow of 1–3% isoflurane in 100% oxygen at a flow rate of 1L/min.
Wipe the skin of the hindlimb with three alternating betadine and alcohol scrubs.
Once the skin is cleaned, obtain one million ESC-derived endothelial cells in 30 μL of phosphate buffered saline (PBS). Load these cells into a 28 gauge needle.
When the cells are ready, gently lift and extend the hindlimb to better visualize the location of the gastrocnemius muscle. While the leg is extended, insert the needle through the skin into the underlying muscle. Take care not to approach the bone. Gently and slowly inject the 30 μL cell mixture into the gastrocnemius. For intramuscular injection, 30 μL is close to the limit of volume that can be injected. Therefore, 28-gauge insulin syringes are preferred because, in our experience, they eliminate the loss volume in syringe needles.
After the injection is complete, return the mouse to the recovery cage and monitor it continuously until awake. Allow the animal to recover for several hours and then proceed with the in vivo bioluminescence imaging of the transplanted cells.

### 4. Bioluminescence imaging of ESC-derived endothelial cells in vivo

The transplanted ESC-derived endothelials cells were modified to express both the fluc and the eGFP fusion gene under the control of an internal ubiquitin promoter. Therefore, bioluminescence can be used to track the cells within the ischemic hindlimb.
To begin this step, turn on the bioluminescence imaging system and Living Image acquisition software. Then initialize the acquisition system and specify the dimensions of the field of view.
Next, place black matte paper onto the imaging box to absorb background emission.
Once the imaging box is ready, place the mouse into the anesthesia induction chamber containing 1–3% isoflurane in oxygen at output of 1L/min. Leave the mouse in the induction chamber until it is unresponsive to external stimuli. Then remove the animal from the induction chamber.
Remove the hair from both hindlimbs using an electric shaver as necessary.
Inject 10 μL of D-luciferin per gram of body weight into the peritoneum. The D-luciferin is prepared in advance into filtered stock solutions of 15 mg/mL in PBS.
Once the luciferin is injected, place the animal into the imaging box over the black paper in the supine position, connected to a continuous flow of isoflurane.
Placing animal into imaging box and connecting to isoflurane.
Start to acquire images for 10–60 seconds in order to determine an optimal exposure time for which the image is not saturated. If the image becomes saturated, reduce the exposure time. If the bioluminescence signal is very weak, increase the exposure time.
Using the optimal exposure time, continue acquiring pictures every 1–3 minutes until the signal reaches the maximum and then fades. When acquisition is complete, save the file.
To analyze the data, select regions of interest (ROIs) that cover the injection site. As a negative control, a similar ROI can be selected for the non-operated leg. Using the software, measure the total radiance, which is expressed in units of photons/seconds/cm^2^/steradian (p/s/cm^2^/sr), for the ROIs for each timepoint. The maximal value should be used in the final data. The data can also be exported to Excel spreadsheet for future use.
Once all the data is acquired, return the animal to the recovery cage and monitor continuously until the animal awakes.
Repeat this procedure to track the cells over time.
At a desired time point, the animal can be euthanized for assessment of tissue function.

### 5. Representative Results


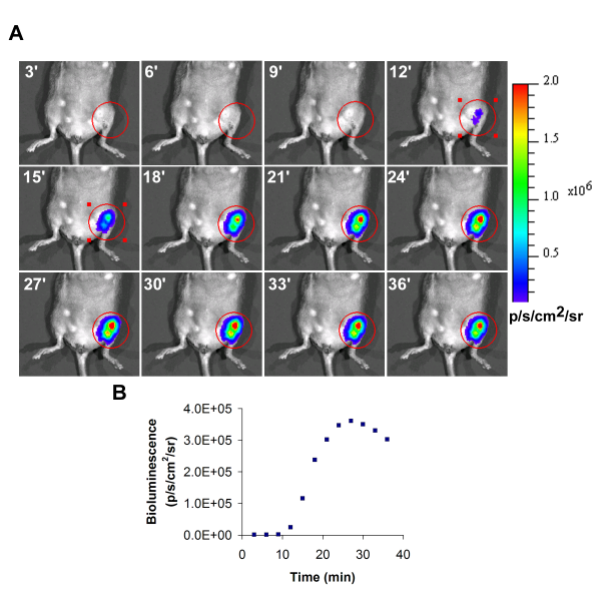


A representative bioluminescence image of transplanted cells in the left ischemic hindlimb is shown in Figure 1. During acquisition of bioluminescence, the intensity will increase with time, and the maximum value obtained during the time course should be reported as the final value.

## Discussion

ESCs are a promising cell source for the treatment of tissue ischemia because of their plasticity of differentiation and their ability to give rise to cell lineages comprising all three germ layers, including endothelial cells. To overcome the ethical concerns associated with ESCs, induced pluripotent stem cells (iPSCs) may be an alternative pluripotent stem cell source that overcomes the ethical concerns. Besides ESCs, adult stem cells such as endothelial progenitor cells (EPCs) and hematopoietic stem cells (HSCs) can also be used, but these cell types may have limited therapeutic effect in patients with PAD. Intramuscular delivery of cells is minimally invasive and easy to perform, and this mode of delivery is also amenable to the delivery of soluble factors or plasmids. However, for easier access of the transplanted cells to the vasculature, systemic delivery into the femoral artery or tail vein can be used instead of intramuscular injections.

Bioluminescence imaging offers the advantage of performing high-throughput and non-invasive tracking of cell survival. When combined with functional assays such as laser Doppler blood perfusion or histological analysis of neovascularization, these techniques together can allow researchers to assess the therapeutic effect of cell transplantation on the recovery of hindlimb ischemia.

In conclusion, we have demonstrated a simple and reproducible method for delivering and tracking of ESC-derived endothelial cells for treatment of hindlimb ischemia.

## References

[B0] Niiyama H, Huang NF, Rollins M, Cooke JP (2008). Murine model of hindlimb ischemia. JoVE.

[B1] Levenberg S, Golub JS, Amit M, Itsakovitz-Eldor J, Langer R (2002). Endothelial cells derived from human embryonic stem cells. Proc. Natl. Acad. Sci. U.S.A.

[B2] Yamashita J, Itoh H, Hirashima M, Ogawa M, Nishikawa S, Yurugi T, Naito M, Nakao K, Nishikawa S (2000). Flk1-positive cells derived from embryonic stem cells serve as vascular progenitors. Nature.

[B3] De A, Yaghoubi SS, Gambhir SS (2008). Applications of lentiviral vectors in noninvasive molecular imaging. Methods Mol Biol.

[B4] Niiyama H, Kai H, Yamamoto T, Shimada T, Sasaki K, Murohara T, Egashira K, Imaizumi T (2004). Roles of endogenous monocyte chemoattractant protein-1 in ischemia-induced neovascularization. J. Am. Coll. Cardiol.

[B5] Cook MJ (1976). The anatomy of the laboratory mouse.

[B6] Contag PR, Olomu IN, Stevenson DK, Contag CH (1998). Bioluminescent indicators in living mammals. Nature Med.

[B7] Ray P, De A, Min JJ, Tsien RY, Gambhir SS (2004). Imaging tri-fusion multimodality reporter gene expression in living subjects. Cancer Res.

[B8] Huang NF, Lee RJ, Li S (2007). Chemical and physical regulation of stem cells and progenitor cells: potential for cardiovascular tissue engineering. Tissue Eng.

[B9] Cao F, Lin S, Xie X, Ray P, Patel M, Zhang X, Drukker M, Dylla SJ, Connolly AJ, Chen X, Weissman IL, Gambhir SS, Wu JC (2006). In vivo visualization of embryonic stem cell survival, proliferation, and migration after cardiac delivery. Circulation.

[B10] Wilson K, Yu J, Lee A, Wu J.C (2008). In vitro and in vivo bioluminescence reporter gene imaging of human embryonic stem cells. Jove.

